# Building climate-resilient, low-carbon health systems: A knowledge, attitudes and practices study with the primary healthcare workforce in Lebanon

**DOI:** 10.1016/j.joclim.2026.100658

**Published:** 2026-02-12

**Authors:** Patricia Nayna Schwerdtle, Adelle Mansour, Kathryn Bowen, Farah Jradi, James Awad, Myriam Mrad, Kelly Carpenter, Sadath Sayeed

**Affiliations:** a Heidelberg Institute of Global Health, Medical Faculty, Heidelberg University, Heidelberg, Germany; b Seed Global Health, Boston, MA, USA; c Melbourne Climate Futures, University of Melbourne, Victoria, Australia; d Melbourne School of Population and Global Health, University of Melbourne, Victoria, Australia; e University of California, Los Angeles, USA; f International Organisation of Migration (IOM), Ramlet Bayda, Lebanon; g University of Balamand Public Health Department, Lebanon

**Keywords:** Climate change, Primary healthcare, Workforce development, Environmental sustainability, Lebanon, Qualitative research, Climate resilience, Low-carbon health systems

## Abstract

**Background:**

Climate change poses increasing threats to human health and health systems, particularly in low- and middle-income countries where exposure and vulnerability tend to be high and readiness to adapt tends to be low. A climate-smart health workforce - equipped to address climate-related health risks and reduce the environmental footprint of healthcare - is a core pillar of resilient health systems. This study explored the knowledge, attitudes, and practices of Lebanon’s primary healthcare workforce concerning climate change and health, to inform capacity-building efforts.

**Methods:**

We conducted a qualitative study using a knowledge, attitudes and practices framework, comprising three focus group discussions (n = 24) with primary healthcare professionals. Data were collected in March 2025, using a semi-structured discussion guide. Thematic analysis was conducted using the Framework Method.

**Findings:**

Participants expressed general awareness of climate-related health risks - particularly respiratory illness and waterborne disease - but demonstrated limited understanding of underlying drivers or systemic impacts. While climate change and health issues were often discussed informally in personal settings, they were rarely integrated into professional practice. Existing sustainability efforts varied across facilities. Nearly all participants reported no prior climate change and health training but expressed strong interest in flexible, practice-oriented learning opportunities.

**Interpretation:**

Despite growing awareness, Lebanon’s primary healthcare workforce lacks structured education and institutional support to respond effectively to climate-related health challenges. Embedding climate change and health competencies into health workforce development - through locally adapted, scalable training programs – is needed to build climate-resilient and low-carbon health systems in Lebanon and similar settings.

## 1 Introduction

Climate change is a profound threat to human health and to the functioning of health systems worldwide. Extreme weather events, shifting disease patterns, slow-onset stressors and environmental degradation are straining health services, especially in regions with fragile health and social systems [1,2]. At the same time, the health sector itself contributes significantly to climate change. In the 2025 Lancet Countdown (Indicator 3.5), the health-care sector accounted for 4.2% of global greenhouse gas emissions, and healthcare-related emissions fell by 12% between 2021 and 2022 following pandemic-related increases [2]. Health systems thus face a dual challenge: they must adapt to protect populations from climate impacts while also mitigating their own carbon footprint [3].

In response, international frameworks and agreements have been established to guide action. The World Health Organization (WHO) has developed an Operational Framework for Building Climate-Resilient and Low-Carbon Health Systems (first released 2015, updated 2023), which outlines ten key components for action. These include a climate-smart health workforce, climate-informed programs, and sustainable financing [4]. This aligns with the global development agenda: United Nations Sustainable Development Goal (SDG) 3 (health) and SDG 13 (climate action), recognizing that progress in health and climate resilience go hand in hand. Indeed, measures that strengthen health systems against climate shocks and stressors while reducing emissions yield co-benefits for both human well-being and environmental sustainability.

Momentum for “climate-resilient, low-carbon” health care has grown rapidly. At the Conference of the Parties (COP) 26 in 2021, 50 countries, including both low-income nations at high climate risk and major emitters, committed to greening and strengthening their health systems [5]. Many pledged steps, such as conducting climate and health vulnerability and capacity assessments and establishing emission reduction targets for the healthcare sector. Today, over 100 countries have made formal commitments, though implementation remains uneven. Initiatives like the WHO-led COP26 Health Programme and the Alliance for Transformative Action on Climate and Health (ATACH) support these efforts by integrating climate-health priorities into national plans and mobilizing resources [6].

Developing a climate-smart health workforce is a key pillar for targeted action, particularly for low- and middle-income countries (LMICs) facing the highest climate-related health risks [4]. Ebi et al. (2021) argue that effective adaptation in health systems hinges not just on infrastructure but on trained, agile personnel capable of anticipating and managing climate-sensitive health risks [7]. Yet many LMICs still lack the foundational workforce training needed for this shift. However, notwithstanding the growing strengths of international commitments to this area, there is limited empirical research on how primary healthcare (PHC) workers in LMICs perceive, engage and respond with climate-related health risks. Assessing the Knowledge, Attitudes, and Practices (KAP) of the health workforce is an important first step towards identifying solutions, such as developing a climate change and health curriculum and integrating it into the training and development of health professionals.

Lebanon’s health system is operating under severe strain from prolonged economic collapse, political instability, and the protracted Syrian refugee crisis, which have eroded infrastructure and human resources [8]. These pressures compound the country’s high exposure to climate hazards such as extreme heat, wildfires, drought, and flooding, amplifying both direct and indirect health risks [9]. In such fragile contexts, building a climate-smart health workforce is not only a matter of long-term resilience but an urgent priority to safeguard service delivery under overlapping crises [10].

**Fig. 1:**
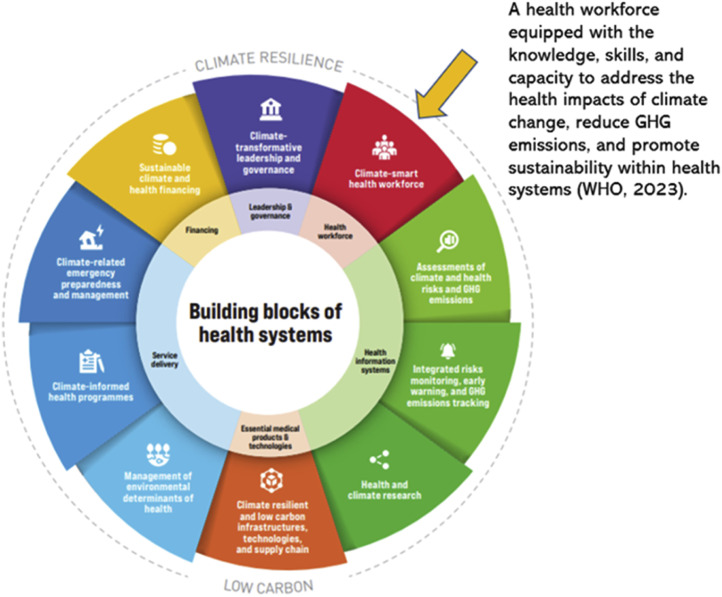
WHO Operational Framework for climate resilient and low carbon health systems focusing on the climate-smart health workforce.

A climate-smart health workforce is made up of health professionals who understand the health impacts of climate change, are equipped to address related challenges, and adopt sustainable practices to help reduce the environmental footprint of healthcare [4]. Recognizing that human resources are central to climate-resilient health systems, the WHO’s Operational Framework (See: Fig. 1) emphasizes workforce development through education and training to manage climate-sensitive health risks. Experts likewise advocate integrating climate-health content across all levels of health education [3]. The 2023 Lancet Countdown report highlights that fewer than 15% of countries have incorporated climate-health education into medical curricula, with particularly low uptake in LMICs [11].

In practice, this involves, for example, clinicians providing guidance on heat-related illnesses and vector-borne diseases, nurses receiving training in responding to climate-related disasters, and administrators integrating sustainability into procurement and day-to-day operation. These competencies are especially important in LMICs, where health workers operate at the frontlines of climate impacts, often in high-risk communities and fragile and under-resourced health facilities [12]. Strengthening the capacities and capabilities of the health workforce enhances clinical care, fosters environmental advocacy, and supports low-carbon innovation in health facilities [13]. As such, the climate-smart workforce serves both adaptation and mitigation goals within health systems.

Health workers around the world increasingly recognize climate change as a serious health issue. A global survey of 4,654 professionals found that most understood climate change is human-caused and a growing threat to health in their country - and many felt a duty to raise awareness with patients and policymakers [13]. Nevertheless, substantial gaps in knowledge and challenges in turning general awareness into pragmatic action within the health workforce remain. Developing effective training programs to help the health workforce anticipate, respond to, and recover from climate-related health shocks and stresses first requires an understanding of their existing knowledge, attitudes, and practices (KAP) related to climate and environmental change and health. KAP studies aim to identify what individuals know (knowledge), believe (attitudes), and do (practices) concerning a specific topic [14].

The aim of this study is to add to the empirical base on how health care workers in LMICs perceive and engage with climate-related health risk, specifically in the country of Lebanon. Existing research on climate change and health in Lebanon appears to have focused on specific environmental health issues such as pollution [15]. To our knowledge, no studies have examined the knowledge, attitudes, and practices (KAP) of Lebanon’s public primary healthcare workforce regarding climate change, environmental sustainability, and health, with a view to inform capacity-building and workforce development initiatives aligned with global climate-health strategies.

## 2 Methods

### 2.1 Study design

We conducted a qualitative study using a KAP framework to explore how Lebanon’s PHC workforce perceives and engages with the health impacts of climate and environmental change. This paper forms part of a broader study exploring both clinical and educational domains. This paper reports the data derived from Focus Group Discussions (FGDs) with primary care health professionals in Lebanon (clinical domain), which was complemented by Key Stakeholder Interviews (KSIs) with health professions educators and curriculum designers (educational domain); findings from the KSIs will be reported separately.

The study sought to generate contextualised, practice-based insights from both clinical and educational domains to inform the design of targeted capacity-building strategies. The research was embedded in a constructivist paradigm and co-designed with local actors, ensuring alignment with in-country priorities and systems.

### 2.2 Setting and participants

The study involved representatives from five public PHC centres in Lebanon, spanning both urban (Beirut, El Matn) and rural regions (Saida, Baalbek, and Tripoli). PHC professionals representing a range of professional groups (e.g., nurses, midwives, social workers, pharmacists) with direct service delivery roles were invited to participate in focus group discussions (FGDs).

### 2.3 Sampling and recruitment

A purposive, stratified sampling approach was used to ensure variation in geographic location, professional role, years of experience, and educational level. Five public primary healthcare centres (PHCCs) were purposively selected in collaboration with the Ministry of Public Health (MoPH) to ensure representation across Lebanon’s main regions and to capture urban–rural, geographic, and socioeconomic diversity. Recruitment was coordinated through MoPH administrative networks rather than self-enrolment to minimise selection bias and ensure variation in professional role and experience. Confirmation bias was mitigated through structured debriefings, team triangulation, and the involvement of bilingual facilitators and analysts to validate interpretations and enhance analytic rigour. FGD participants (n = 24) were licensed PHC professionals currently working in public centres and involved in direct patient care. Recruitment was facilitated via Ministry of Public Health (MoPH) administrative networks. Sampling continued iteratively until thematic saturation was reached, meaning that no new themes emerged across successive focus group discussions, and key concepts were consistently repeated across diverse participant subgroups, indicating adequate depth and breadth of data to address the study objectives. Thematic saturation was determined when no new codes or concepts emerged in the final focus group discussion (FGD3) and when patterns identified in earlier FGDs were consistently repeated across participants from different regions and professional backgrounds.

### 2.4 Data collection

Data were collected between 19 March and 31 March 2025. A total of three FGDs were held (6–10 participants each). Two FGDs were conducted in Arabic and one in English, based on participant preference; no simultaneous interpretation was required. FGDs were facilitated by trained local researchers, using a semi‑structured question guide derived from the KAP framework (see Supplementary Material). The guide included questions and prompts exploring participants’ knowledge of climate‑related health impacts, attitudes toward climate resilience and sustainability, climate‑smart practices in healthcare delivery, and training needs. The question guide included follow-up prompts to explore causal linkages between climate change and health, such as its effects on nutrition, mental health, and non-communicable diseases. Each FGD lasted approximately 45–60 minutes. Data were recorded through structured note‑taking and summary sheets and were supported by post-session debriefing. Audio recording was not used at the request of local partners and participants, reflecting both political sensitivities and a preference for a less formal setting to encourage open discussion.

### 2.5 Translation and transcription procedures

The FGD question guide, explanatory statement, and consent form were translated from English into Arabic by bilingual members of the in‑country research team. Translations were reviewed collaboratively to ensure conceptual equivalence, cultural appropriateness, and clarity prior to field use. As no audio recordings were made, no verbatim transcripts were produced; instead, structured note‑taking tools served as the primary dataset. Structured note-taking tools consisted of summary templates developed prior to data collection, with clearly defined sections aligned to the focus group discussion questions. These templates were used to systematically capture key discussion points, illustrative quotes, and observational notes, supporting consistency and transparency across focus groups. Bilingual facilitators and note‑takers recorded points of discussion directly in English or Arabic and the notes were later translated into English for analysis.

### 2.6 Structured debriefing and reflexive integration

Shortly after the FGDs were conducted, research team members conducted structured debriefing sessions with facilitators and note‑takers, drawing on the systematic debriefing method that advocates goal‑oriented review immediately following qualitative encounters to enhance data quality and support preliminary analysis. These debriefs were used to clarify meanings, reconcile ambiguous content, and identify possible themes across the dataset [16]. The local research team brought deep contextual knowledge, and their reflections during debriefing enriched interpretation and facilitated early analytic insights. This process generated a supplementary dataset that informed and shaped subsequent coding and framework analysis, thus strengthening trustworthiness, enhancing reflexivity, and initiating analysis in real time.

### 2.7 Data analysis

Data were analysed using the Framework Method [17] to allow for systematic comparison within and across datasets. A deductive-inductive coding approach was used: data were initially indexed using codes derived from the KAP framework, with additional codes identified during the coding process. NVivo software (QSR International) was used for data management and coding. A subset of transcripts were independently double-coded to enhance reliability. Structured debriefing notes from the FGDs were integrated into the analytic process to contextualise findings and refine interpretation; we then charted the data and examined matrices to identify themes and patterns. The analytic process was iterative, with frequent team discussions to examine deviant cases and achieve interpretive depth.

### 2.8 Ethical considerations

Ethical approval for the study was obtained from the Ethics Committee at Université Saint-Joseph de Beyrouth (reference: CER-2025-111), in coordination with the Lebanese Ministry of Public Health (MoPH) and the International Organization for Migration (IOM). All participants provided written informed consent. Consent was reconfirmed verbally at FGD start and end, and confidentiality expectations were reviewed. No financial incentives beyond transport reimbursement were provided. Confidentiality and data protection protocols were strictly followed. Participation was voluntary, and participants were informed of their right to withdraw at any time without consequence. Logistical support, including transport reimbursement and flexible scheduling, was provided to facilitate participation.

## 3 Results

**Fig. 2:**
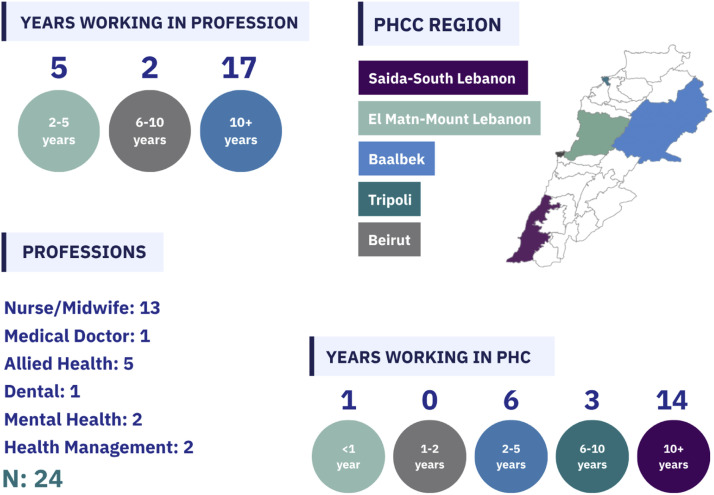
Demographic information of the sample.

We conducted three FGDs with 24 PHC professionals from five public health centres across Lebanon (Fig. 2). Thematic analysis identified eight themes within the KAP framework. A detailed table of themes, subthemes, and full illustrative quotes is provided in Table 1.

**Legend**Fig. 2. This figure summarizes the demographic distribution of focus group participants. Most participants were nurses or midwives (n = 13), followed by allied health professionals (n = 5), health management staff (n = 2), mental health workers (n = 2), and one medical doctor and one dental practitioner. The majority had over 10 years of professional experience (n = 17) and more than 10 years working in primary healthcare (n = 14). Participants were drawn from five public primary healthcare centres (PHCC) across Lebanon: Saida–South Lebanon, El Matn–Mount Lebanon, Baalbek, Tripoli, and Beirut.

**Table 1:** Themes, subthemes and additional illustrative quotes.

	Theme	Subthemes	Illustrative Quotes
Knowledge	1. Awareness of Climate Change-Related Health Impacts and Vulnerable Populations	Observed changes in weather, agriculture, and water Surface-level awareness of vulnerable groups	*“Climate change has contributed to a rise in wildfires across Lebanon. It has also negatively impacted nutrition, as fruits and vegetables are increasingly contaminated by polluted water”* (Pharmacist Assistant, FGD1) *“Climate change for me is something that’s happening all over the world, it’s a global change. If we look at the past years, for example, the winters were colder than they are now, the temperatures weren’t like this. There’s a clear change happening compared to before”* (Psychology Department Coordinator, FGD2) *“Those most vulnerable to infections are individuals with weaker immune systems, including children and the elderly”* (Pharmacist Assistant, FGD1)
	2. Perceived Link Between Climate Change and Disease Burden	Increase in respiratory and infectious diseases Climate seen as a direct cause	*“There is an increase in respiratory tract diseases, and illnesses are recurring more frequently than before”* (Doctor, FGD1) *“During the winter season, flu cases rise. In the past, we simply called it the flu, but now doctors give it more specific names. The change in climate has had a profound impact on diseases. Fluctuations in temperature and daily temperature changes have significantly contributed to the spread of the flu”* (Nurse, FGD3)
Attitudes	3. Climate Resilience and Environmental Sustainability as Shared Responsibility	Individual and institutional responsibility Government role and infrastructure deficits	*“Responsibility is shared by all of us. We must manage electricity and water consumption, medical supplies, and, when necessary, seek out alternative solutions”* (Nurse, FGD3) *“Additionally, the government shares responsibility for enacting and enforcing these policies”* (Midwife, FGD3)
	4. Adaptation as Practical and Operational	Focus on improving water infrastructure Tangible, actionable suggestions	*“Infrastructure problem in Lebanon is a big issue. Wastewater is mixing with the water in the Litani River, and the decline in rainfall is preventing the polluted water from being flushed out. This is leading to increased contamination, unpleasant odors, and a significant accumulation of waste”* (Doctor, FG1) *“Upgrading infrastructure is essential to prevent polluted water from mixing with water used for food consumption” (Quality Coordinator, FGD1)*
Practices	5. Climate Awareness in Personal, Not Professional, Conversations	Climate discussed with family/friends Rarely raised with patients or at work	*“Yes, we frequently discuss climate change with family and friends”* (Dental Assistant, FGD1)
	6. Environmental Sustainability Varies Across Facilities	Uneven practices: waste sorting, quality control, solar energy	*“In our practice, we have a quality control system to ensure proper sorting of products. Since we are accredited, strict quality control is in place”* (X-ray Professional, FGD1) *“Previously, medical waste was burned, but now it is being refined*” (Nurse, FGD1)
Training	7. Lack of Training and Strong Desire for Capacity Building	Absence of prior climate change and health training Willingness to engage in future opportunities	*“We haven’t done any trainings before”* (Registered Nurse, FGD2) *“We must have complete knowledge of the information in order to work properly”* (Psychology Department Coordinator, FGD2)
	8. Training Preferences and Priorities	Preference for flexible formats Focus on climate-health linkages in training content	*"We would love for you to visit us in Baalbek to provide workshops and awareness sessions."* (Medical Doctor, FGD1)

### 3.1 Participant characteristics

Of the 24 FGD participants, 20 were female and 4 were male. Participants represented diverse professional roles, including nurses (n = 12), social workers (n = 2), midwives (n = 1), doctors (n = 1), and others such as mental health workers, educators, and technical staff. Coordinators were licensed health professionals holding managerial roles. Most participants held higher education qualifications, including TS (Technicien Supérieur), LT (Technical License Diploma), and master’s degrees. Seventeen participants had over 10 years of professional experience, and 14 had over 10 years of experience specifically in PHC settings. Participants were distributed across five regions: Saida (n = 8), Baalbek (n = 8), Tripoli (n = 4), Beirut (n = 3), and El Matn (n = 1). Key findings are presented below and thematically summarised in Table 1.

### 3.2 Knowledge of climate, environment, and health linkages

**Theme 1: Awareness of climate change-related health impacts and vulnerable populations**

Participants demonstrated an experiential understanding of changing weather patterns and environmental impacts, often noting observable shifts in rainfall, humidity, and temperatures. Vulnerable populations were occasionally mentioned, with limited explanation of underlying reasons.

*“The people most vulnerable to climate change are the poor, children, the elderly, pregnant women, and those with chronic conditions.”* (Director, FGD2)

**Theme 2: Perceived link between climate change and disease burden**

Climate change was frequently linked to an increased burden of respiratory and infectious diseases. Participants described a worsening pattern of symptoms and healthcare demand attributed to climatic shifts.

*“We see more allergies and respiratory issues because climate change is impacting the chest and causing lung problems.”* (Nurse, FGD3)

### 3.3 Attitudes towards climate resilience and environmental sustainability

**Theme 3: Climate resilience and sustainability as shared responsibility**

Participants viewed climate action as both an individual and institutional obligation, calling on government agencies to lead by example.

*“As healthcare professionals, it is our duty to lead by example in implementing these measures.”* (Doctor, FGD1)

**Theme 4: Adaptation as practical and operational**

Adaptation was framed in pragmatic terms, with emphasis on infrastructure improvements, particularly in water and sanitation.

*“More effort should be directed toward improving infrastructure to prevent contamination of clean water with wastewater.”* (X-ray Professional, FGD1)

### 3.4 Practices in climate-smart healthcare delivery

**Theme 5: Climate awareness embedded in personal, not professional, conversations**

Climate and environmental topics were often discussed in personal contexts, but rarely integrated into patient care.

*“We talk about it often, especially about how Lebanon no longer experiences four distinct seasons.”* (Nurse, FGD1)

**Theme 6: Environmental sustainability varies across facilities**

Sustainability practices ranged from structured waste sorting and solar energy adoption to minimal or inconsistent measures.

*“Transitioning from fossil fuels to solar energy is imperative for promoting sustainability.”* (Nurse, FGD3)

### 3.5 Training and capacity building needs

**Theme 7: Lack of training and desire for action**

Most participants had no prior climate change and health training but expressed strong willingness to engage in learning opportunities.

*“We have never had any training or awareness sessions on this topic.”* (X-ray Professional, FGD1)

**Theme 8: Training preferences and priorities**

Preferred formats included blended learning (online and in-person), with climate-health linkages as a core focus.

*“Both online modules and in-person awareness sessions would be beneficial.”* (Pharmacist Assistant, FGD1)

## 4 Discussion

This study is the first to report on the Lebanese public primary health care workforce’s knowledge, attitudes, and practice regarding climate change and its impact on health. Overall, our findings suggest that participants possess a basic awareness of climate change impacts on health, a professional sense of responsibility for addressing its impact, and interest in structured training to acquire complementary skills for their practice.

Studies from other countries share some similarities with these findings. For example, in South Africa, health workers expressed strong interest in environmental sustainability and saw it as part of their professional role. Respondents there displayed incomplete understanding of the indirect negative health effects of climate change, such as its link to malnutrition [18]. In India, over 80% of surveyed clinicians and community health workers recognized the health risks of heat, cold, and vector-borne diseases, with many eager to learn more [19]. In both studies, respondents felt unsure how to translate their concerns into practice, citing limited training and resources; “green” practices were only minimally adopted. A 2024 survey in U.S. primary care clinics, largely serving low-income communities, found that while 84% of staff saw climate change already affecting patients, only 36% discussed it in consultations, often due to time constraints [20]. Over half reported they were motivated to learn how to help patients prepare for extreme weather events [20].

We are aware of only a few studies conducted in the Middle East, and these have primarily focused on nursing student populations [21,22]. Similar to findings from South Africa, India, and the United States, participants in our study recognised climate change as a pressing health issue. However, their understanding was often limited to observable weather patterns (such as extreme heat or cold) and immediate health effects like respiratory illnesses, with less attention given to indirect or systemic impacts, such as the spread of vector-borne diseases, disruptions to health service delivery, or the mental health effects of displacement and economic stress. Vulnerable populations were occasionally mentioned; however, participants did not identify refugee or displaced populations (highly relevant in Lebanon) as climate-vulnerable groups. This omission reveals the need to strengthen equity-oriented climate-health training, including the interconnections between climate change, migration/displacement and health and the migrant-inclusiveness in addition to the climate resilience of primary healthcare systems. Participants expressed a strong sense of responsibility to act, consistent with global literature describing health workers as willing climate advocates [13]. However, few translated this concern into professional practice, often citing structural barriers, such as political instability, inadequate infrastructure, and administrative overload, that limit their capacity to engage with climate-health issues, even when awareness is present. Participants expressed strong motivation but also uncertainty about how to act, citing limited institutional guidance and resources. This reflects a confidence gap rather than attitudinal resistance.

As in other settings, climate change, environment and health discussions were largely confined to private spheres, rarely integrated into clinical consultations or workplace protocols, suggesting a more abstracted link between climate change and the need to build pragmatic skills and competencies to lessen its impact on patient health outcomes [20].

A key gap identified was the absence of structured training on the interconnections between climate, environment, and health. While participants expressed interest in flexible and accessible learning modalities, aligning with global shifts toward module and self-paced education, they reported limited access to formal opportunities for building relevant competencies. This lack of exposure to formal curricula may help explain at least some of the knowledge and practice gaps observed in our findings. To enable healthcare professionals to meaningfully engage with complex and evolving health impacts of climate change, training should be deliberate, prioritized and embedded within professional education frameworks [23].

Similar challenges have been documented elsewhere. Blom et al. (2025) emphasised that achieving Kenya’s net-zero health system ambitions will require developing a climate-smart and environmentally sustainable health workforce. They found that awareness of the health sector’s contribution to climate change was generally low and that sustainability principles were not yet integrated into pre-service or in-service training. Stakeholders highlighted the importance of incorporating climate and environmental sustainability into health education, fostering behavioural and cultural change among staff, and linking training and awareness efforts to policy guidance, incentives, and accountability mechanisms. Strengthening workforce capacity and leadership was identified as a key enabler for translating national climate and sustainability commitments into action within health facilities [24].

In practical terms, climate change and health training for PHC workers in Lebanon could be delivered through blended formats that combine self-paced online modules with in-person workshops embedded within existing continuing professional development (CPD) structures. Linking climate change and health competencies to recognised accreditation pathways, such as the Lebanese Order of Nurses or Ministry of Public Health professional licensure requirements, would help ensure uptake and sustainability. Integration into regular PHC quality improvement cycles could further normalise climate-health practice as part of routine service delivery. These recommendations also align directly with the WHO ATACH commitments, which call for climate-resilient and low-carbon health systems through workforce strengthening. Implementing context-specific climate change and health training in Lebanon could serve as a model for other fragile and conflict-affected settings facing overlapping climate and health system stressors.

Importantly, climate change and health toolkits, curricula, and competency frameworks are increasingly available [23]. However, their integration into pre- and in-service education remains inconsistent in many countries, especially LMICs due to substantial resource constraints [3]. Effective implementation requires both prioritization by relevant ministries and adequate resourcing. In some high-income countries, where climate-health content has been introduced, curricula coverage remains uneven [25]. While momentum is building to embed climate change into health professional education, few interprofessional curricular initiatives have been developed or rigorously evaluated. Nonetheless, existing studies indicate such approaches can improve climate-health knowledge and foster collaborative practice skills [26].

To ensure training effectiveness, a structured monitoring and evaluation (M&E) framework should accompany climate change and health capacity-building initiatives. This could include pre- and post-training assessments of knowledge and skills, longitudinal follow-up to assess integration into practice, and facility-level indicators for climate-smart service delivery. Such evaluation would help refine training content and support evidence-based scale-up.

Strengths of this study lie in its use of a qualitative research design and the involvement of in-country partners as co-authors/researchers. Qualitative methods enable in-depth exploration of phenomena. Indeed, extant literature increasingly advocates for combining qualitative approaches with more traditional, quantitative approaches to assessing KAP [19,27]. Through data collection methods such as focus groups and interviews, one can capture health workers' subjective experiences, uncover barriers, and identify practical solutions. Moreover, the contextual knowledge and expertise provided by in-country partners notably helped to strengthen the interpretation and applicability of findings herein.

This study has several limitations. It focused on individual-level knowledge, attitudes, and practices among a subset of primary healthcare professionals in Lebanon and does not fully represent the views of all PHC workers, despite purposive sampling across roles, regions, and facility types. Grouping five PHC centers into three focus group discussions limited the ability to compare findings by center or region. Time and resource constraints also prevented examination of institutional readiness to integrate climate change and health into PHC. Additionally, the absence of audio recordings and reliance on structured note-taking may have reduced the capture of nuance, particularly in translation from Arabic to English. The research team mitigated this through careful phrasing and contextual clarification in analysis discussions with bilingual members. Given the small, geographically constrained sample (n=24), findings represent an exploratory snapshot rather than a comprehensive assessment of Lebanon’s PHC workforce. Nonetheless, the study offers context-specific insights that can inform targeted inquiry and programmatic adaptation in similar settings.

Future research could build on this exploratory qualitative study through a nationwide survey to more systematically assess KAP and training needs related to climate change and health across the PHC workforce. This could be complemented by a curriculum mapping study to identify where climate change and health topics are currently addressed in pre-service and in-service training, and where integration could be strengthened. Future work should also focus on piloting and evaluating climate change and health training programs in PHC settings, measuring their impact on clinical practice and patient outcomes, and scaling effective models across Lebanon and other fragile, climate-vulnerable contexts. In addition, mixed-methods and intervention studies are needed to evaluate the effectiveness of such training initiatives and assess institutional readiness for implementing climate-resilient and low-carbon practices in Lebanon’s primary healthcare system.

## 5 Conclusion

Our research offers a novel and timely contribution to what is known about health care providers’ understanding of and experience with climate and environmental change and its health implications in Lebanon, an LMIC. This study highlights the importance of workforce-focused interventions as part of national climate-health strategies. As Lebanon continues to face compounding challenges - including economic instability and forced migration - a climate-smart PHC workforce is needed to ensure equitable and sustainable health system resilience. Significant barriers, including limited resources, expertise, and regulatory mandates, impede expressed interest in building out a climate-smart workforce in Lebanon. The need to add more intersectoral resources to develop targeted training and context-specific learning materials, and integration of climate change and health content into both pre-service education and continuing professional development, is clear. Strengthening institutional mandates is likely an essential next step to advance a successful and coordinated effort. Future work should focus on piloting and evaluating climate change and health training programs in PHC settings, measuring their impact on clinical practice and patient outcomes, and scaling effective models across Lebanon and other fragile, climate-vulnerable contexts.

## Funding

This research was funded by the Philippines - Department of Foreign Affairs.

## Data availability statement

De-identified qualitative data supporting the findings of this study are available from the corresponding author upon reasonable request, subject to ethical and institutional approvals in line with agreements with the Lebanese Ministry of Public Health and the International Organization for Migration.

## CRediT authorship contribution statement

**Patricia Nayna Schwerdtle:** Writing – review & editing, Writing – original draft, Visualization, Validation, Supervision, Resources, Project administration, Methodology, Investigation, Formal analysis, Data curation, Conceptualization. **Adelle Mansour:** Writing – review & editing, Validation, Methodology, Investigation, Formal analysis, Data curation, Conceptualization. **Kathryn Bowen:** Writing – review & editing, Project administration, Funding acquisition, Conceptualization. **Farah Jradi:** Writing – review & editing, Validation, Project administration, Methodology, Investigation, Data curation, Conceptualization. **James Awad:** Writing – review & editing, Validation, Project administration, Investigation, Data curation. **Myriam Mrad:** Writing – review & editing, Validation, Investigation, Conceptualization. **Kelly Carpenter:** Writing – review & editing, Visualization, Validation, Resources, Project administration, Funding acquisition, Conceptualization. **Sadath Sayeed:** Writing – review & editing, Writing – original draft, Validation, Supervision, Resources, Project administration, Methodology, Funding acquisition, Conceptualization.

## Declaration of competing interest

The authors declare that they have no known competing financial interests or personal relationships that could have appeared to influence the work reported in this paper.

## Figures and Tables

**Table:** 

TOPIC	QUESTIONS
**A. Knowledge of Climate and Environmental Change***(incl. health impacts)*	1. **What comes to mind when you hear the term ‘climate change,’ and how have you experienced or observed its effects in your environment and community?** (Prompt: Can you share any specific stories or changes you’ve noticed in weather patterns, daily life, or environmental degradation?) 2. **In what ways do you think climate change is already impacting HEALTH, both in general and in your community?** (Prompt: What specific health issues have emerged or worsened? Who do you think is most vulnerable? 3. **Looking ahead, what concerns you most about climate-related health risks, and what actions or solutions do you think are needed to build climate resilience?** (Prompt: Can you think of examples of climate-resilient solutions or actions?)
**B. Attitudes Toward Climate Resilience and Environmental Sustainability**	4. **How important do you think climate resilience and environmental sustainability are, both generally and in the context of primary health care?** (Prompt: First, let’s think about climate resilience. What about environmental sustainability? Why do these concepts matter, and how do they relate to your work?) 5. **What role should the primary healthcare sector play in implementing climate-resilient and environmentally sustainable solutions?** (Prompt: What do you see as your own role in this? What challenges or opportunities do you anticipate?)
**C. Climate-Smart Practices in Health Care Delivery**	6. **What sort of climate and environmental issues come up in your work, and in what context?** (Prompt: What topics do you discuss, how do these conversations arise, and with whom—patients, colleagues, or others?) 7. **What environmentally sustainable practices are currently in place at your facility, and how effective are they?** (Prompt: Who is responsible for implementing them, and what is your role?) 8. **What challenges have you encountered in reducing the environmental impact of your facility, and what more could be done to improve these practices?** (Prompt: Can you share examples of difficulties and opportunities for further action?)
**D. Training and Capacity Building Needs**	9. **Have you received any training on climate change, its health impacts, or environmental sustainability in your healthcare work?** (Prompt: What topics were covered, and in what format?) 10. **What kind of training or skills would help you better respond to climate change in your healthcare facility?** (Prompt: What format would be most useful—workshops, online modules, etc.?)
**Close**	11. **Would anyone like to add anything that we have not already discussed?** 12. **Do you have any questions for me?**
